# Diagnostic posture control system for seated-style echocardiography robot

**DOI:** 10.1007/s11548-022-02829-3

**Published:** 2023-03-07

**Authors:** Yuuki Shida, Masami Sugawara, Ryosuke Tsumura, Haruaki Chiba, Tokuhisa Uejima, Hiroyasu Iwata

**Affiliations:** 1 Graduate School of Creative Science and Engineering, Waseda University, Tokyo, 169-8050 Japan; 2 Global Robot Academia Laboratory, Waseda University, Tokyo, 169-8050 Japan; 3 NSK Ltd Technology Development Department 1New Field Products Development Center Technology Development Division Headquarters, Kanagawa, 251-8501 Japan; 4 The Cardiovascular Institute, Tokyo, 106-0031 Japan; 5 Faculty of Science and Engineering, Waseda University, Tokyo, 169-8050 Japan

**Keywords:** Medical robots, Echocardiography, Robotic ultrasound, Human–robot interaction

## Abstract

**Purpose:**

Conventional robotic ultrasound systems were utilized with patients in supine positions. Meanwhile, the limitation of the systems is that it is difficult to evacuate the patients in case of emergency (e.g., patient discomfort and system failure) because the patients are restricted between the robot system and bed. Therefore, we validated a feasibility study of seated-style echocardiography using a robot.

**Method:**

Preliminary experiments were conducted to verify the following two points: (1) diagnostic image quality due to the sitting posture angle and (2) physical load due to the sitting posture angle. For reducing the physical burden, two unique mechanisms were incorporated into the system: (1) a leg pendulum base mechanism to reduce the load on the legs when the lateral bending angle increases, and (2) a roll angle division by a lumbar lateral bending and thoracic rotation mechanisms.

**Results:**

Preliminary results demonstrated that adjusting the diagnostic posture angle allowed to obtain the views, including cardiac disease features, as in the conventional examination. The results also demonstrated that the body load reduction mechanism incorporated in the results could reduce the physical load in the seated echocardiography. Furthermore, this system was shown to provide greater safety and shorter evacuation times than conventional systems.

**Conclusion:**

These results indicate that diagnostic echocardiographic images can be obtained by seated-style echocardiography. It was also suggested that the proposed system can reduce the physical load and guarantee a sense of safety and emergency evacuation. These results demonstrated the possibility of the usage of the seated-style echocardiography robot.

## Introduction

Heart disease has become the most common disease globally in terms of deaths. According to a World Health Organization (WHO) [1] survey, 17.9 million people died of heart disease in 2019. The mortality rate of heart disease can be significantly improved by early detection and treatment. In response to this situation, transthoracic echocardiography often referred to as cardiac ultrasound has become the modality of choice in the initial assessment of cardiac disease because it is noninvasive, easy to use, and provides high-resolution imaging and real-time feedback [2]. However, ultrasonography, including transthoracic echocardiography, is highly challenging because of its complex procedure. As a result, physicians and sonographers are required to have high experience and knowledge.

**Fig. 1:**
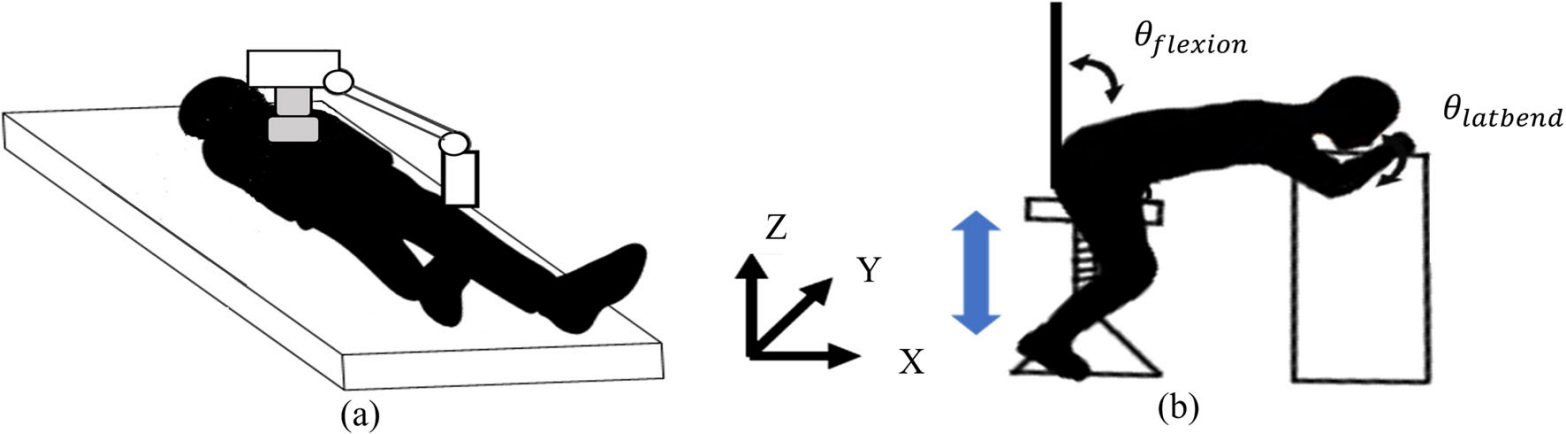
Echocardiography robot and subject placement **a** supine, **b** seated

To solve the problems mentioned above, a wide range of robot-assisted technologies for ultrasound examinations have been developed. These robot systems have focused on several applications such as the shoulder [3], carotid artery [4], liver [5], fetal [6–8], cardiac tamponade [9], transesophageal echocardiography [10], echocardiography [11], and other generic sites [12, 13]. However, most of these robot systems are designed to be used with the patient in the left lateral decubitus or supine position, which is the recommended position for conventional examination methods. As representative cases, Ref [5] developed a robotic system for automatic ultrasound imaging focusing on human liver. Reference [9] proposed robotic ultrasound-guided facet joint insertion. Reference [11] developed a support system for handling ultrasound probe to alleviate fatigue of physician by introducing a coordinated motion with robot. Those configurations were mainly applied through a serial robotic manipulator or gantry-style (see Fig. 1a). In the case that the robot performs the examination in that position, the patient is positioned between the robot and the bed. Therefore, due to the restricted posture of the patient, emergency evacuation in case of patient discomfort or robot failure is difficult. This could be insufficient for safety during the examination. Therefore, it is ideal that the patient undergoes the examination in a sitting position rather than in the left lateral decubitus or supine positions in terms of safety and emergency evacuation because the patient can immediately leave the robot system (see Fig. 1b). In the conventional examination, the patient needs to be in the supine position first, and the examination is performed in the left lateral decubitus position when diagnostic images cannot be obtained clearly since the heart is hindered by other organs such as the lungs. By examining in the left lateral supine position, the heart can be slightly shifted. By doing so heart will not remain hindered by other organs. This is achieved by adjusting the direction of gravity applied to the heart. We hypothesize that the same phenomenon can be produced by adjusting the angle of the patient’s posture, even in the sitting posture. If the quality of diagnostic images is ensured in the sitting posture, the safety of the robot system for assisting the ultrasonography can be guaranteed.

Therefore, the purpose of this study is to establish the proof-of-concept of the seated-style echocardiography robot system. We experimentally analyzed the diagnosable sitting posture angle at which diagnostic images visualizing the features of cardiac diseases can be acquired with healthy subjects. Based on the analysis, we proposed a sitting posture control system for the seated-style echocardiography that enables the adjustment of the optimal sitting posture angle, thereby visualizing the features of cardiac disease and decreasing the physical load occurring in the sitting position. For verifying the proposed system, we evaluated the decrease of physical load by introducing the system. Also, we performed the user’s subjective evaluation in terms of comfortability and safety and measured the evacuation time compared to the conventional spine-style robot system.

## Method

### Design requirement

In order to develop the seated-style echocardiography robot, it is necessary to determine its design requirements. Meanwhile, since the echocardiography in the sitting position is not commonly performed to date, it is questionable whether it is possible to acquire ultrasound images with high diagnostic quality in the sitting position and what is the appropriate posture angle of patients for the image acquisition. Therefore, two preliminary tests are preformed to determine the design requirements: (1) diagnostic image quality due to the sitting posture angle and (2) physical load due to the sitting posture angle.

#### Diagnostic image quality due to sitting posture angle

In this preliminary test, we verify whether it is possible to acquire ultrasound images with features required for the diagnosis of cardiac diseases in the sitting posture and investigate the image quality due to the sitting posture angle. According to a clinical expert in the field of echocardiogram, observation of three features is necessary to diagnose myocardial infarction, valvular disease, and cardiomegaly, which are the major diseases observable by echocardiography (1) ventricular motion, (2) dynamics and shape of the valve, and (3) enlargement of the ventricular wall. We first acquired the parasternal long-axis and apical four-chamber views. These are the basic diagnostic views used to extract those thee features. After the acquisition, assessment is conducted to check if three features are recognizable in two basic views, when the posture angle changes. With six healthy subjects, those views were acquired by a clinical expert in the field of echocardiography. Each view was acquired in the left lateral decubitus position and sitting posture with ten conditions. A medical ultrasound system (EPIQ7, Philips, Netherland) and a matrix array sector probe (X5-1, Philips, Netherland) were used for the ultrasound image acquisition. An optical tracking sensor (Trio V120, OptiTrack, Japan) attached to the ultrasound probe was used to measure the position and angle of the ultrasound probe. A diagnostic posture angle adjustment table was used to change the angle of the sitting posture. The detailed flow of the experiment is described below.

**Fig. 2:**
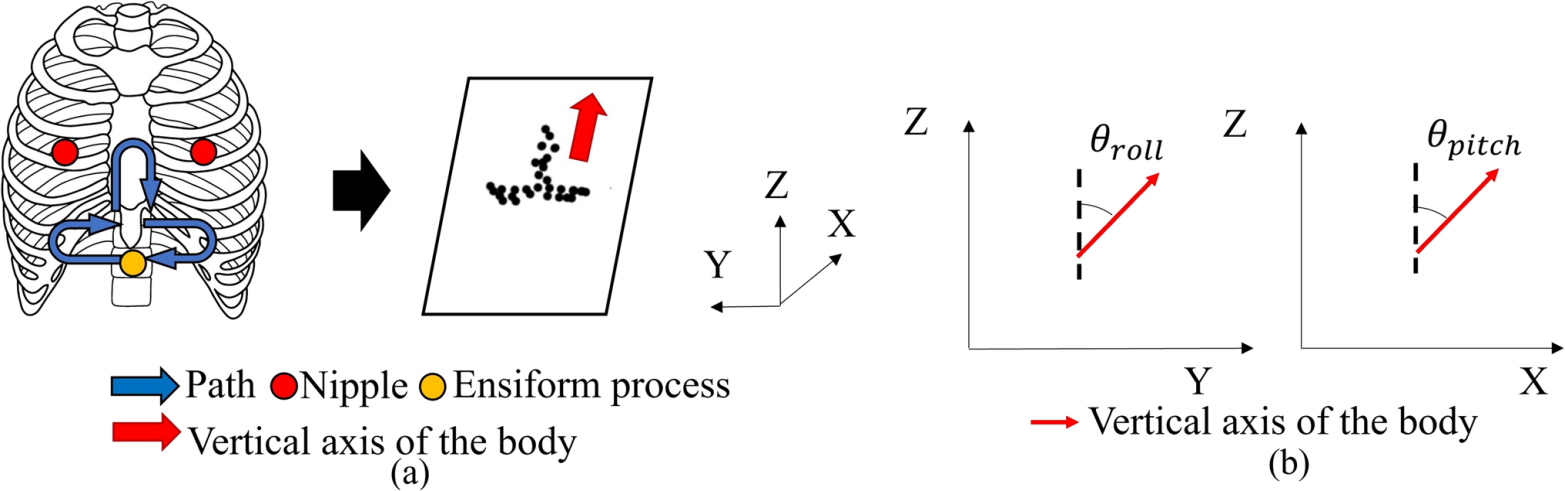
Marker tracking calibration. **a** Probe travel path and plane approximation; **b** calculation of *θ*_roll,_ *θ*_pitch_

**Fig. 3:**
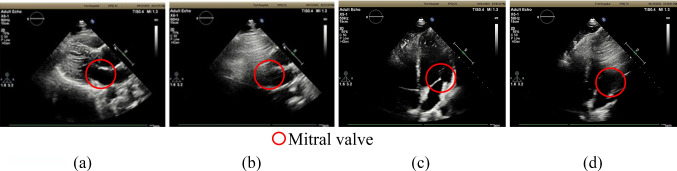
Ultrasound images in sitting posture; **a** clear parasternal long-axis view; **b** unclear parasternal long-axis view; **c** clear apical four-chamber view; **d** unclear apical four-chamber view

1. The subject is placed in the left lateral decubitus position. Then, images are acquired in the parasternal long-axis view and the apical 4-chamber view.
2. The subject is placed in the sitting posture and on the diagnostic posture angle adjustment table. As shown in Fig. 2a, following process were conducted with probe tracking to obtain the actual patient’s posture angle: (i) with the ensiform process as the starting point of the probe scanning, the probe was moved left and right and up and down to the nipple position; (ii) planar approximation is applied to the tracked probe positions, and then the actual posture angle of the subject was calculated. As shown in Fig. 1b, in this study, *X*-axis is defined as sagittal axis of the body when standing vertically. The *Y*-axis is defined as forehead axis of the body. The *Z*-axis is defined as longitudinal axis of the body. Note that *θ*_roll_ and *θ*_pitch_ are defined as shown in Fig. 2b.
3. The probe is moved to the position where the parasternal long-axis view and the apical four-chamber view can be acquired. Then, the subject stops breathing, and ultrasound images are acquired for two seconds while the subject is holding his breath. Each view is acquired three times.
4. The subject’s sitting posture is in all nine conditions of the flexion angle *θ*_flexion_ (approx. 30°, 60°, 90°) in addition to the lateral bending angle *θ*_latbend_ (approx. 0°, 15°, 30°), as shown in Fig. 1. We follow the same procedure given in (1) to obtain the parasternal long-axis and apical four-chamber views.
5. The physician scores each view on a three-point scale (diagnostic superiority, undecidable, and nondiagnostic quality) in terms of its ability to visualize the three aforementioned features. Based on this evaluation, the number of visible cardiac features is used to identify the sitting posture to ensure the image quality. Figure 3 presents representative ultrasound images with diagnostic superiority and nondiagnostic quality acquired in the sitting posture.

**Fig. 4:**
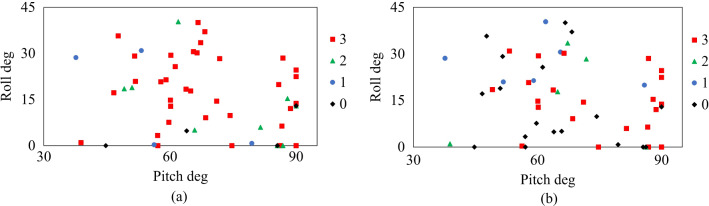
Relationship between the number of diagnosable features and sitting posture angles. **a** The number of diagnosable features and posture angles in the parasternal long-axis view; **b** the number of diagnosable features and posture angles in the apical four-chamber view

**Fig. 5:**
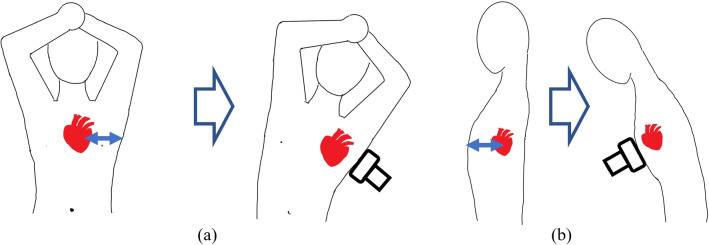
Change of heart position during body posture change; **a** roll; **b** pitch

Figure 4a, b shows the relationship between the number of visible cardiac features and sitting posture angle in the parasternal long-axis and apical four-chamber views. From these results, it can be seen that it is possible to obtain each view where the three features can be detected even in the sitting posture. In the parasternal long-axis view, it was suggested that all the target disease features could be depicted in a posture with a roll angle of 10°–30° and a pitch angle of 50°–80°. On the other hand, in the apical four-chamber view, it was suggested that all the features of the target disease could be captured in a posture with *θ*_roll_ = 10°–20° and *θ*_pitch_ = 60°–70°. We assume that as shown in Fig. 5a, b, when the body posture is angled along the direction of roll and pitch, the distance between the heart position and the probe becomes closer, which may prevent echo attenuation by the lungs. This may be thought to make it easier to acquire the clear image. On the other hand, as the roll and pitch angles were too inclined, the number of diagnosable features of the heart decreased. This could be because the heart had more contact with the thoracic wall by increasing the posture angle than when the patient was in the left lateral supine position used in the usual examination. This could have made the heart challenging to move and observe, which was observed in the apical four-chamber view rather than the parasternal long-axis view. As a result, apex of the heart observed in the apical four-chamber view is closer to the thoracic wall than any other part of the heart, and the postural angle causes the apex to contact the thoracic wall more quickly, affecting the motion of the heart. In this study, we analyzed the data of six subjects, and each of them had a different diagnosable posture angle, possibly due to the individual differences in heart position. According to medical knowledge [14], heart position varies among individuals and is classified into three types: right-leaning, center, and left-leaning. A left-leaning heart is closer to the thoracic wall than a right-leaning one. Therefore, the shift of the heart position due to posture variation is reduced. This difference is thought to cause individual differences in postural angles that can be diagnosed.

#### Physical load due to the sitting posture angle

In the previous section, we investigated the image quality in terms of cardiac feature visualization due to the sitting posture angle. Conversely, some posture angles may impose a physical burden on the patient, making it difficult to perform the examination for a long time. In this preliminary test, the relationship between flexion and lateral bending angles in the sitting posture of patients and their physical load is verified by subjective evaluation using VAS. Based on the verification results, we derive the design requirements for a sitting posture control system for the seated-style echocardiography that enables patients to undergo the examination without any physical burden.

**Fig. 6:**
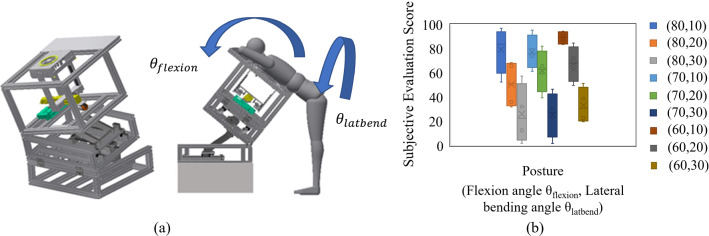
**a** Posture support mechanism for verification of body load in sitting test, **b** relationship between subjective assessment of physical load and posture angles

In this preliminary test, we used a support jig (see Fig. 6a) to adjust the flexion and the lateral bending angles. First, the subject’s flexion angle *θ*_flexion_ (60, 70, and 80°) and lateral bending angle *θ*_latbend_ (10, 20, and 30°) were varied in all nine conditions. Next, six subjects were asked to make a subjective evaluation with VAS on whether they could maintain their posture sufficiently. The relationship between posture and physical load is analyzed based on the results. In the subjective evaluation, the case where the physical load was large, and it was challenging to maintain the posture was evaluated as zero, and the case where the posture could be maintained sufficiently was evaluated as 100.

The relationship between the subjective evaluation of physical burden and diagnostic posture is shown in Fig. 6b. The results suggest that *θ*_latbend_ is more responsible for the physical burden of the subject than *θ*_flexion_. First, the range of motion for lateral bending of the thoracolumbar region is smaller than for flexion. Second, the greater the lateral bending angle, the larger the angle between the ground and leg, and the more the load is applied to the foot base. Also, as the lateral bending angle increases, the angle between the ground and the leg increases, and the load is placed on the foot base. As a result, the force to support the body is concentrated on the leg in the lateral bending direction, and the load is considered to increase.

Based on these results of preliminary tests, we found the feasibility of seated-style echocardiography, and the necessity to support the sitting posture at the optimal angles in terms of the image quality and physical burden of patients. Then, we propose a posture angle control system to realize the seated echocardiography robot in the next section.

### Design concept

The preliminary results in the previous section showed the feasibility of seated-style echocardiography by adjusting the sitting posture angle. Meanwhile, the condition visualizing the cardiac features in the sitting position depended on individual differences, and some sitting posture angles caused the physical load on the patient. Therefore, the function that adjusts the sitting posture angle is needed for the seated echocardiography robot.

**Fig. 7:**
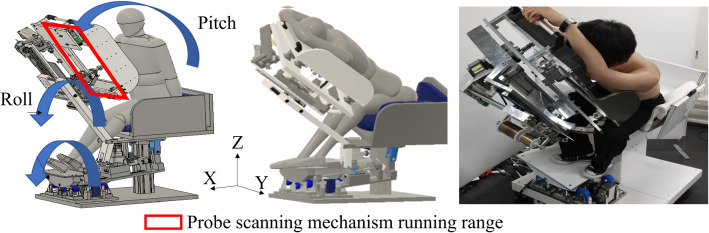
Seated posture angle control system

**Fig. 8:**
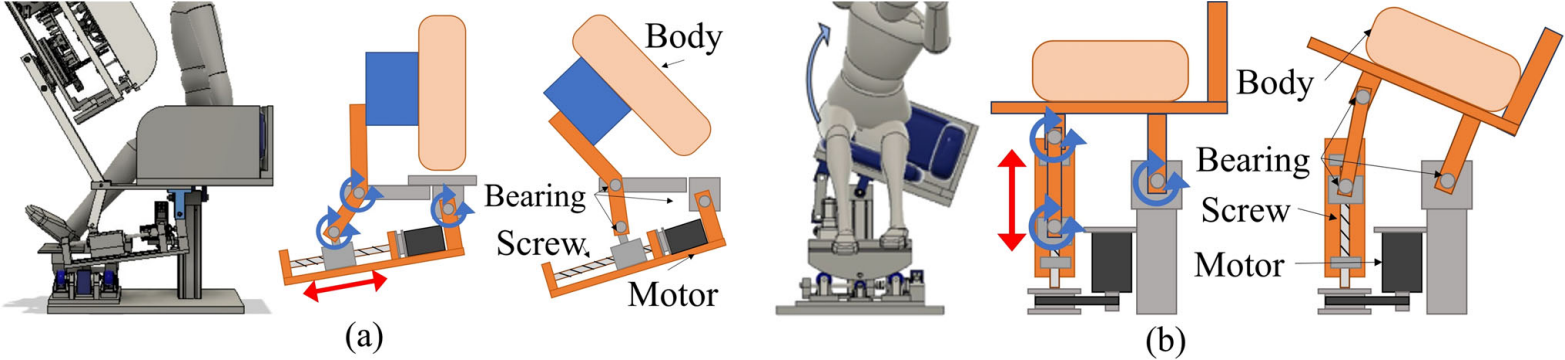
**a** Lumbar flexion mechanism, **b** lumbar lateral bending mechanism. Note that red and blue arrows indicate each figure’s active and passive motion parts

**Fig. 9:**
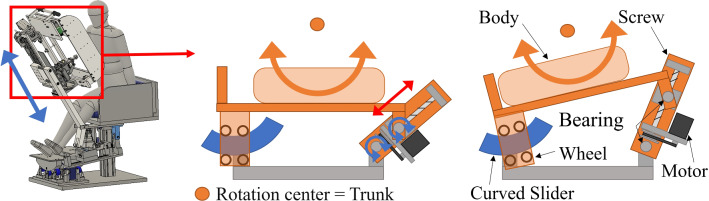
Thoracic rotation mechanism. Note that red and blue arrows indicate each figure’s active and passive motion parts

The developed system consists of three active degrees of freedom (DOFs) and two passive DOFs to control the patient body at any posture angle while reducing the physical load on the patient (see Fig. 7). The pitch angle of the posture is controlled by a lumbar flexion mechanism using a four-section linkage mechanism, a trapezoidal screw thread (MTSTRW16-475-F38-V12-S60-Q12, MISUMI, Japan), and a stepper motor (RKS5913RAD2-3, ORIENTAL MOTOR, Japan). As shown in Fig. 8a, the lumbar flexion mechanism is operated by moving the trapezoidal screw thread in the direction of the red arrow. The upper body support unit is connected to the trapezoidal screw unit via the rotating shafts, so that the translation motion with the trapezoidal screw is converted to rotation motion of pitch angle. The roll angle of the posture is controlled by two mechanisms: a lumbar lateral bending mechanism (see Fig. 8b) using a trapezoidal screw thread (MTSBRV16-232-F38-V12-S70-Q12-C30-J0, MISUMI, Japan) and a stepper motor (RKS5913RAD2-3, ORIENTAL MOTOR, Japan), and a thoracic rotation mechanism (see Fig. 9) using a trapezoidal screw thread (MTSBRV16-322-F32-V10-S65- Q12-C60-J3, MISUMI, Japan) and a stepper motor (RKS599RAD2-2, ORIENTAL MOTOR, Japan). The rotational motion of the roll angle is achieved by combining these mechanisms, the details of which are explained in the following section. In the preliminary test, the subjects were six males in their twenties, and the variation in the experimental conditions was limited. Considering the differences in age and gender, the differences in diagnosable posture angles due to individual differences could be even more considerable. Then, the required angle in this system is *θ*_roll_ = 0°–65° and *θ*_pitch_ = 0°–85°. The thoracic rotation mechanism can be manually moved in the direction of the blue arrow shown in Fig. 9 by self-weight compensation using constant load springs (CR-16.CR-19, Accurate, Japan). This allows the user to adjust the height to suit their height manually. In terms of the safety, as all the DOFs are achieved by the linear motions with the trapezoidal screws, the system maintains its posture even if the motor stops, thus ensuring patient safety in case of emergency. It also provides sufficient space for adding the probe scanning mechanism (see Fig. 7). This space is 250 mm in the direction of the body vertical axis, 200 mm in the direction of the body forehead axis, and 350 mm in the direction of the body sagittal axis. This was determined experimentally based on average chest width and heart size. Additionally, to reduce the physical burden during the examination, the system is equipped with two mechanisms (1) a leg pendulum base mechanism to reduce the burden on the legs when the lateral bending angle is increased, and 2) a roll angle division by the lumbar lateral bending mechanism and the thoracic rotation mechanism.

### Leg pendulum base mechanism

**Fig. 10:**
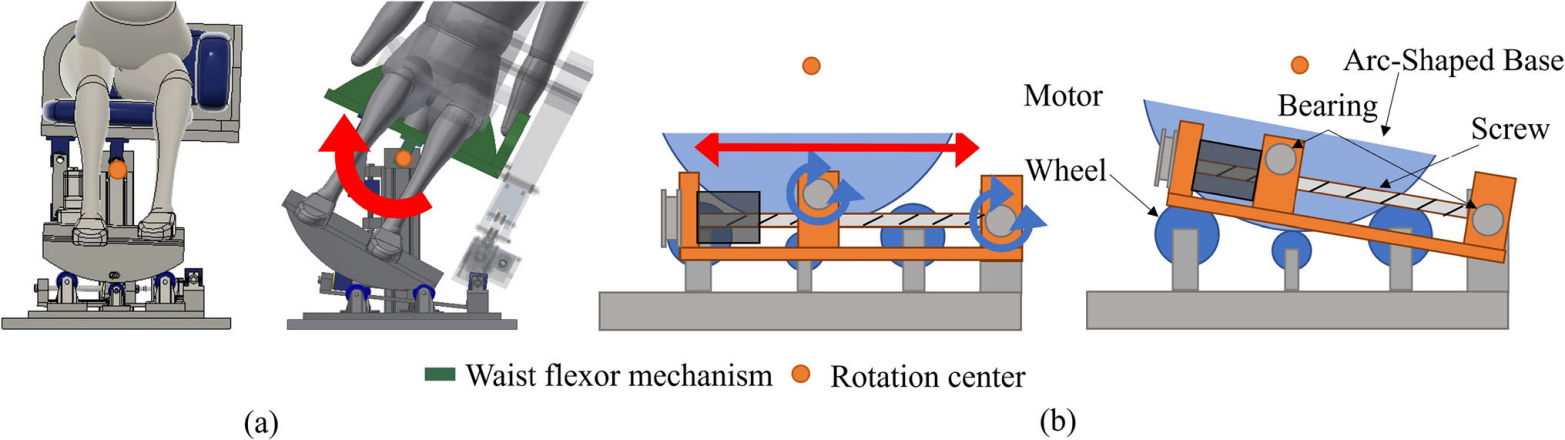
Leg pendulum base mechanism; **a** mechanism operation; **b** mechanism details. Note that red and blue arrows indicate each figure’s active and passive motion parts

This mechanism can change its angle in synchronization with the lateral bending angle of the lumbar lateral bending mechanism described in the previous section to maintain the legs and base vertical. As shown in Fig. 10b, the base with an arc at the bottom is supported by three wheels. The base rotates smoothly according to the side-bending angle. The base moves by a linear motion mechanism using a trapezoidal screw thread (MTSTLW16-307-F30-V12-S51-Q12, MISUMI, Japan) and a stepper motor (RKS566RAD2-3, ORIENTAL MOTOR, Japan). As shown in Fig. 10a, the center of rotation is shifted above the base. This allows the base to move in an arc relative to its center of rotation and the base can behave like a pendulum movement. By making the rotation centers of the waist lateral bending and this mechanism identical, the distance between the two mechanisms can be kept constant even when the lateral bending angle is increased while the waist/leg installation parts of the two mechanisms remain parallel. This allows the user to maintain a stable posture without having the legs lift off the base or having a mid-back posture when the lateral bending angle changes.

### Lumbar lateral bending mechanism and thoracic rotation mechanism

The leg pendulum base mechanism can reduce the cause of the physical load described in the previous section. Alternatively, a force must be applied to the legs to withstand the force of the gravity component applied in the inclined direction of the base, resulting in a foot load. The roll angle is induced by combining the lumbar lateral bending and thoracic rotation mechanisms to solve this problem. This reduces the tilt angle of the lumbar lateral bending mechanism/leg pendulum base mechanism to minimize the body load.

Moreover, the lumbar lateral bending and thoracic rotation mechanisms have been devised to enable lateral bending and rotation with minimal body load. As shown in Fig. 8b, the lumbar lateral bending mechanism is operated by moving the trapezoidal screw thread in the direction of the red arrow. As the gluteal support unit is connected to the trapezoidal screw thread unit, the translation motion with the trapezoidal screw is converted to rotation motion of roll angle at the lumbar. The thoracic rotation mechanism allows the upper body to rotate around the trunk axis of the upper body. As shown in Fig. 9, this is achieved by connecting the upper body support unit to a curved slider with four wheels and a linear motion mechanism with trapezoidal screw threads via the rotating shafts. As a result, the connection can be tilted when the linear motion mechanism is moved while the center of rotation is adjusted to the trunk axis.

## Evaluation

### Experimental setup

To validate the developed diagnostic posture control system, the following two experiments were conducted: (1) efficacy of the physical load reduction mechanism, and (2) comparison between the conventional supine-style and seated-style configurations.

#### Efficacy of physical load reduction mechanism

In this experiment, we quantitatively evaluate the physical load on each muscle when using the proposed posture angle control system. The effectiveness of reducing the physical load by applying the leg pendulum base mechanism and dividing the roll angle using the lumbar lateral bending and thoracic rotation mechanisms is also tested. The quantitative evaluation of the body load is performed using a muscle potential measurement device (Wireless EMG system Trigno, 4 Assist, Japan). The number of subjects was 6 (height: 172 ± 16 cm; weight: 62 ± 9 kg). The detailed flow of the experiment is described below.

1. The EMG analyzer is attached to the gastrocnemius and oblique abdominal muscles on each side.
2. In the four conditions listed in Table 1, the subject’s posture is kept at a *θ*_*g_*roll_ of 20° and *θ*_*g_*pitch_ of 45° for two minutes. The EMG in each part of the body at that time is recorded. Note that *θ*_*g_*roll_ is 90° minus the angle between the gravity vector and the frontal axis, and *θ*_*g_*pitch_ is 90—the angle between the gravity vector and the sagittal axis.
3. A band-pass filter with a cutoff frequency of 20 Hz and 450 Hz is applied to the EMGs acquired under each condition, and the signal envelope using the root mean square value of a 300 ms moving slit is extracted. Note that this process was performed with reference to Ref. [15].
4. The median of the values calculated in (3) is computed, and this value is used as the measured EMG in each condition. Next, the sum of the measured EMG of the left and right gastrocnemius and oblique abdominal muscles in each condition is calculated as the leg and abdominal loads, respectively. Then, conditions A and B in Table 1 are compared to verify the effectiveness of the leg pendulum base mechanism; conditions B, C, and D are compared to verify the effectiveness of reducing the physical load by dividing the roll angle by the lumbar lateral bending and thoracic rotation mechanisms; and conditions A and D are compared to verify the effectiveness of combining the two physical load reduction mechanisms. Note that the low EMG value demonstrates the low physical load.

**Table 1:** List of conditions for verification of body load reduction mechanism

Condition	Leg pendulum base mechanism	Roll (°)	
		Lumbar lateral bending mechanism	Thoracic rotation mechanism
A	Not introduced	20	0
B	Introduced	20	0
C	Introduced	0	20
D	Introduced	10	10

#### Comparison between conventional supine-style and seated-style configurations

**Fig. 11:**
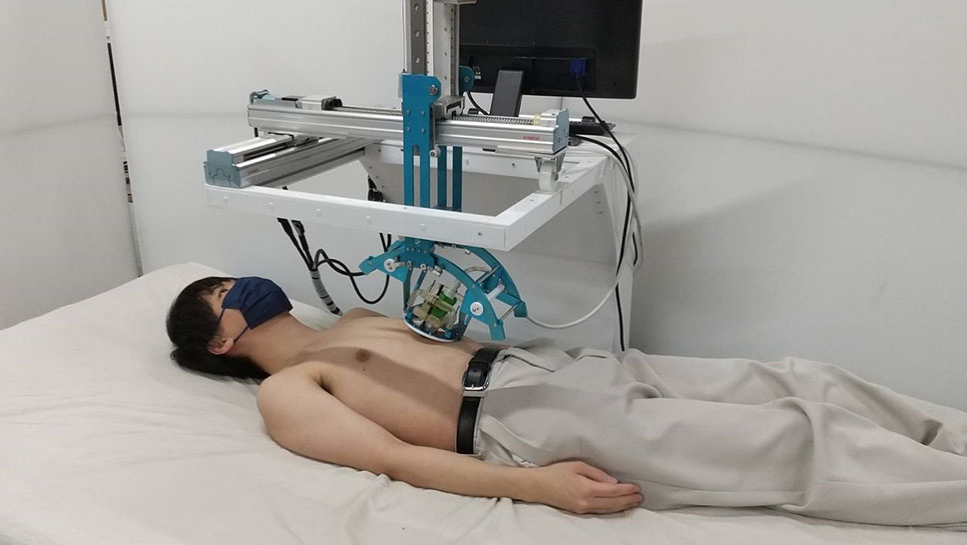
The experiment setup of supine-style configuration [16]

This experiment compared the comfortability, safety, and evacuation time between the conventional ultrasound robot configuration of supine-style and the proposed seated-style configuration. As the supine-style configuration system, the previous developed robotic ultrasound system [16] shown in Fig. 11 was used. The number of subjects was 6 (height: 179 ± 9 cm; weight: 64 ± 7 kg). The detailed flow of the experiment is described below.

1. The subject was placed in the supine position on a bed or seated on the posture control system. In the spine position, the subjects were not tilted. The posture angle of the seated subject was kept at *θ*_*g_*roll_ of 20° and *θ*_*g_*pitch_ of 45°.
2. The subject is placed with the probe attached to the xiphoid process of the chest. From this state, the subject dismounts from the robot and stands on the ground. The time at that point is measured and used as the evacuation time.
3. The subject is asked to rate the comfortability and safety of each system on a 5-point scale (low: 1 to high: 5).

### Results

#### Efficacy of physical load reduction mechanism

**Fig. 12:**
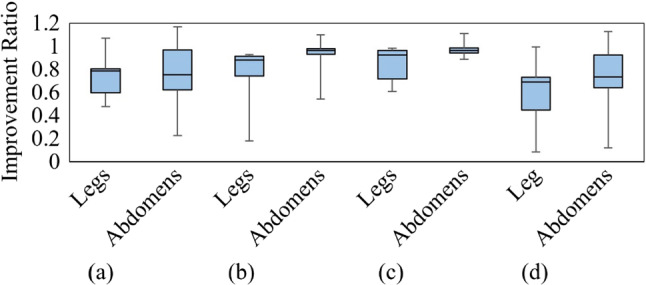
Ratio of leg/abdominal load for each condition; **a** comparison of the use of leg pendulum base mechanism (conditions A vs. B); **b** comparison of lumbar lateral bending mechanism only and a roll angle division by lumbar lateral bending mechanism and thoracic rotation mechanisms (conditions B vs. D); **c** comparison of thoracic rotation mechanisms only and a roll angle division by lumbar lateral bending mechanism and thoracic rotation mechanisms (conditions C vs. D); **d** comparison of the use of two body load reduction mechanisms (conditions A vs. D)

Figure 12 shows the ratio of leg and abdominal load for each condition. The median ratio of leg/abdomen load comparing of the use of leg pendulum base mechanism (conditions A vs. B in Fig. 12a) was 0.785 for the legs and 0.754 for the abdomen. Additionally, the median ratio of leg/abdomen load comparing of lumbar lateral bending mechanism only and a roll angle division by lumbar lateral bending mechanism and thoracic rotation mechanisms (conditions B vs. D in Fig. 12b) was 0.879 for the legs and 0.965 for the abdomen. The median ratio of leg/abdomen load comparing of thoracic rotation mechanisms only and a roll angle division by lumbar lateral bending mechanism and thoracic rotation mechanisms (conditions C vs. D in Fig. 12c) was 0.924 for the legs and 0.964 for the abdomen. Lastly, the median ratio of leg/abdomen load comparing of thoracic rotation mechanisms only and a roll angle division by lumbar lateral bending mechanism and thoracic rotation mechanisms (conditions A vs. D in Fig. 12d) was 0.691 for the legs and 0.736 for the abdomen.

#### Comparison between conventional supine-style and seated-style configurations

**Fig. 13:**
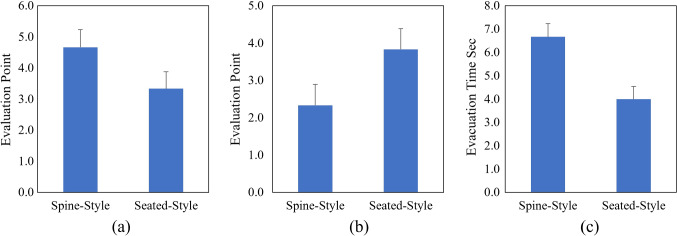
Comparison of subjective evaluation between the spine-style and seated-style configurations in terms of **a** comfortability, **b** safety, and **c** evacuation time

The results of the comparison between the spine-style and seated-style configurations are shown in Fig. 13. The average comfortability scores were 4.7 in the supine-style and 3.3 in the seated-style; the average safety scores were 2.3 in the supine-style and 3.8 in the seated-style; the average evacuation time was 6.7 s in the supine-style and 4.0 s in the seated-style.

### Discussion

#### Efficacy of physical load reduction mechanism

The results of the previous section suggested that the leg pendulum base, lumbar lateral bending, and thoracic rotation mechanisms effectively reduced the body load by dividing the roll angle. On the other hand, it was found that some subjects disagreed with the effectiveness of these mechanisms in reducing body load due to individual differences in the muscles of the human body. For example, if the leg strength is superior to the abdominal muscles, the load may be less in condition B than in condition D. Therefore, it would be possible to reduce the load on the body by providing an appropriate ratio of the load on the trunk, leg strength, and abdominal muscles for each individual.

#### Comparison between conventional supine-style and seated-style configurations

The results of the previous section suggest that the subjective safety and evacuation time in the seated-style superior to in supine-style, while the comfortability in the seated-style was inferior. Since the subject is not covered by the system in case of the seated-style configuration, the improvements of subjective safety and the evacuation time make sense. On the other hand, the seated posture with the current system is more physically demanding than the spine posture, and thus may be less comfortable. This seems to be due to the problem of body axis bending caused by the rotational moment due to the body’s gravity during body lateral bending. The bending of the body axis is considered to cause physical strain because the patient must use muscle strength to control the bending of the body axis and maintain the posture, which potentially causes a lack of comfort. To solve this problem, we recognize to incorporate a mechanism in this system to support the force of the rotational moment caused by the body’s gravity. 

#### Limitations

The limitations of this study are discussed following. First, the physical loads were only evaluated for the short period and needed to be assessed over a longer period. We will investigate the relationship between the physical load measured with EMGs and the difficulty of maintaining the posture angle for a long time. The second limitation is that the posture angle control system was developed based on the limited number of subjects. There may be subjects who cannot be examined in the sitting posture due to individual differences. We recognize it is necessary to perform a comparative study on a more significant number of subjects with variations in gender, body size, and age. The third limitation is that the effects on the obtained image due to the change of the examination position need to be more investigated. Although it has been confirmed that the necessary diagnostic features can be observed in the sitting posture, there might be some other effects. For example, the position and expanding way of the lung may be changed due to the examination posture. This could increase the lungs coverage of the heart and makes it difficult to obtain a clear image. In some cases, the change of the examination posture may alter the direction of the load on the heart due to gravity, which compresses the heart and affects the pulsation. In addition, although this study verified whether the cardiac features can be observed in the seated position by a single physician, it is necessary to increase the number of physicians for investigating their variation of visibility.

Finally, this system does not yet incorporate a system for manipulating the probe, and then it has not been verified as to whether this system can acquire ultrasound images with the diagnosable quality as same as the preliminary results in the previous section. In order to obtain the ultrasound image, the position and contact pressure of the probe needs to be adjusted sophisticatedly under satisfying its safety. Although the current system cannot evaluate the safety regarding the probe manipulation, we assume to use several previously proposed methods for ensuring the safety, such as applying an admittance control [7, 17] or introducing an end-effector with passive-actuated mechanism [18]. It is also necessary to compare spine-style and separated-style systems in terms of the quality of acquiring ultrasound images. These limitations will be verified in the future by introducing the probe manipulation mechanism to this system.

## Conclusion

This manuscript presents a feasibility study of seated-style echocardiography. Conventional robotic ultrasound systems were utilized with patients in supine positions since their configurations are mostly serial robotic arm and gantry-style. Meanwhile, the limitation of the systems is that it is difficult to evacuate the patients in case of emergency (e.g., patient discomfort and system failure) because the patients are restricted between the robot system and bed. Then, it is ideal that the patient undergoes the examination in the sitting position in terms of safety. Preliminary results showed that adjusting the diagnostic posture angle enabled us to obtain the views, including cardiac disease features, as in the conventional examination. Also, the results showed that a physical load occurred for patients depending on their posture angle. Based on those results, a seated posture control system was proposed to adjust the sitting posture without causing any physical load during the examination. Two unique mechanisms to reduce the physical load were incorporated into the system: (1) a leg pendulum base mechanism to reduce the load on the legs when the lateral bending angle increases, and (2) a roll angle division by a lumbar lateral bending mechanism and a thoracic rotation mechanism. Experimental results demonstrated that those mechanisms could reduce the body load, which occurred in the seated echocardiography. In addition, it was demonstrated that the robot has a higher sense of security and a shorter evacuation time than conventional robots. These results showed the potential of the seated-style echocardiography robot. In the future, we plan to apply the posture angle control system and the ultrasound probe scanning mechanism to automatic echocardiography using robots.
